# Evaluation of a learning module on team competencies in medicine utilizing simulation-based scenarios

**DOI:** 10.1186/s12909-026-09274-9

**Published:** 2026-04-24

**Authors:** Lea Herberg, Franziska Körner, Florian Junne, Kristina Geue

**Affiliations:** 1 Department of Psychosomatic Medicine and Psychotherapy, Medical Faculty , University Clinic of Psychosomatic Medicine and Psychotherapy, Otto-von- Guericke-University Magdeburg, Leipziger Str. 44, Magdeburg, 39120 Germany; 2 German Center for Mental Health (DZPG), Partner Site Halle-Jena- Magdeburg, Magdeburg, Germany; 3 Center for Behavioral Brain Sciences (CBBS), Magdeburg, Germany

**Keywords:** Healthcare teams, Collaboration, Interprofessional education, Curricula models, Role-playing, Feedback

## Abstract

**Background:**

Interprofessional collaboration is essential for effective healthcare delivery, yet its integration into medical education remains challenging. Interprofessional education (IPE) has the potential to enhance patient care, mutual respect, and teamwork skills, but barriers such as resistance to change and limited curricular integration persist. The primary objective of this evaluation study is to contribute to the development and integration of IPE into medical curricula, with an outlook of addressing global healthcare challenges and enhancing patient care.

**Methods:**

A newly developed IPE module at the University Medical Center Magdeburg was evaluated. The module focuses on enhancing team competencies through clinical simulation and debriefing. Data were collected using a standardized paper-pencil questionnaire with quantitative items addressing structural, didactic, and content-related aspects, measured on a 5-point Likert scale (1 = very satisfied to 5 = dissatisfied). The survey was administered to all seminar participants after completing the learning module. Descriptive and inferential statistical analyses, including linear regression, were conducted to examine relationships between variables.

**Results:**

A total of 197 medical students (29.8% male, 70.2% female, mean age 23.8 ± 3.5 years) participated in the learning unit. Participants expressed overall satisfaction with the seminar (M = 1.95, SD = 0.95). Key aspects such as moderation by the lecturer, atmosphere, and student involvement were highly rated (69–71%), while simulation-based learning scenarios showed the greatest variance in evaluations. Regression analysis showed that interest in interprofessionalism significantly predicted overall satisfaction (β= -0.337, *p* < 0.001, R²= 0.127) and perceived learning outcomes (β= -0.336, *p* < 0.001, R²= 0.113).

**Conclusions:**

The findings demonstrate the module’s effectiveness in fostering interprofessional attitudes, with high student satisfaction and engagement. Suggested improvements include aligning simulation topics with clinical interests and enhancing feedback processes, such as incorporating video feedback. Strategies to increase situational interest and address low engagement among some students include integrating novel learning elements and implementing structural changes. This study underscores the potential of simulation-based learning in preparing medical students for effective collaboration within complex clinical environments.

**Supplementary Information:**

The online version contains supplementary material available at 10.1186/s12909-026-09274-9.

## Introduction

Interprofessional collaboration holds immense potential to benefit all stakeholders in healthcare, yet its realization remains a complex and demanding task [1]. The integration of interprofessional education (IPE) into healthcare curricula is widely advocated as a means to foster collaborative practices, improve patient outcomes, and prepare future professionals for interdisciplinary teamwork. Recent studies emphasize the critical role of interprofessional identity in this context. Reinders and Krijnen (2023) demonstrate that stronger interprofessional identification correlates with increased interprofessional effort [2].

Despite its promise, developing a sustainable framework for interprofessional education continues to face significant barriers. Key challenges include overcoming resistance to change, aligning IPE with institutional goals, and embedding interprofessional competencies into existing educational structures [3, 4].

To address these challenges, it is crucial to understand the concept of interprofessional collaboration, which is defined as the direct cooperation of professionals from different occupational groups, each with distinct specializations, professional self- and external perceptions, areas of competence, fields of activity, and varying statuses [5]. The goal of such collaboration is to achieve a complementary, high-quality, patient-centered care by leveraging the specific competencies of each profession to optimally benefit the patient. Both the World Health Organization (WHO) and the Interprofessional Education Collaborative (IPEC) emphasize the necessity of integrating interprofessional education throughout the entire medical curriculum. This integration - from early didactic experiences to advanced clinical practice - is crucial for achieving meaningful outcomes and addressing the complex challenges faced by the global healthcare system [6, 7].

Recent literature underscores the urgent need for systematic implementation and evaluation of IPE interventions. Scoping reviews by Cadet et al. [8] and Jiang et al. [9] report that IPE significantly enhances key aspects of patient care, including reductions in medical errors, improved patient education, and shorter hospital stays [8, 9]. However, a systematic review of 15 international studies examining the effectiveness of IPE for medical and nursing professionals highlighted limitations in the robustness of the evidence base and the scarcity of studies [10]. Saragih et al. [11] further confirm that interprofessional learning modules play a significant role in enhancing attitudes and mutual respect towards other healthcare professions [11].

Another scoping review has shown that clinical simulation is an effective method for training healthcare professionals in conflict management by enhancing interprofessional collaboration, teamwork, and communication skills [12]. Despite its positive impact on clinical practice and healthcare organization, further research is needed to validate its application across different contexts, as the review’s limited number of studies underscores the need for more comprehensive research in this area. Effective team competencies are crucial for improving patient safety and healthcare outcomes [13].

To address these challenges and contribute to the research gap, a new interprofessional learning module was developed and implemented at the University Medical Center Magdeburg. The module is designed to span an academic year and incorporates a blend of lectures and a practice-oriented seminar. The seminar involves simulation scenarios, role-playing and structured debriefing. Furthermore, the module is characterized by a clearly defined curricular placement within the medical program—a level of detail that is frequently lacking in existing IPE research [14]. This study evaluates the learning module to ensure quality and continuous improvement based on student feedback. It seeks to address the following research questions:

1. How do students evaluate the content and didactics of the completed learning module on interprofessionalism?
2. What relationships can be identified between students’ personal interest in interprofessionalism, the anticipated importance of interprofessionalism in future professional practice, and the evaluation of the learning module?
3. How does interest in interprofessional collaboration and anticipated importance of interprofessionalism influence the satisfaction with the learning outcomes in theory and practice?

## Methods

### Study design

A total of 197 third-year medical students participated in the interprofessional learning unit. They were taught in 20 small-group courses (7–12 students) during April and May of the 2024 summer semester. At the end of each seminar a cross-sectional survey with a self-developed standardized paper-pencil questionnaire on structural, didactic and content-related framework conditions was handed out to explore satisfaction with the new learning unit, interest in and the expected importance of interprofessional collaboration and its interrelationships. All participating third-year medical students were required to provide written informed consent before taking part in the study. They then completed the questionnaire on a voluntary basis to contribute to the improvement and further development of the learning unit. The approval of the ethics committee of the Medical faculty was obtained (66/24).

### Description of the learning module

The teaching concept consisted of two lectures (135 min in total) and a small group seminar (135 min). While the first lecture provided a basic introduction to the topic and conveyed evidence-based knowledge, the seminar focused on simulated but realistic situations through simulation-based learning scenarios.

In the first exercise, students reflected on their team roles (according to Belbin) and discussed the importance of these roles for professional collaboration [15]. This was followed by an introduction to feedback rules to ensure constructive feedback processes. The first scenario simulated an emergency patient handover using the SBAR scheme (Situation, Background, Assessment, Recommendation), followed by self-reflection, reflection of others, and feedback in small groups. The second simulation-based learning scenario focused on an interprofessional ward round in which the roles of physician, medical student, nurse, social worker and patient were taken. The scenario simulated and reflected the perspectives of each professional group in the decision to discharge an elderly patient. The debriefing was conducted by experienced instructors with expertise in medical communication, who also served as the seminar’s moderators. Finally, students discussed the transferability of the interprofessional rounds to everyday clinical practice and reflected their contribution in promoting interprofessional collaboration in their future work context. A detailed description of the teaching unit can be found in Geue et al. (2025) [16].

### Measurements

The self-developed standardized paper-and-pencil questionnaire collects demographic data from students and measures their satisfaction with various aspects of the seminar. The questionnaire is comprised of a total of 15 items. The initial 13 items evaluate satisfaction with the structure (outline, organization), the theoretical and practical learning outcome, the teacher’s moderation, the use of simulation-based learning scenarios, the selection of scenario topics, the ratio of theory and practice, the level of performance expectations, the learning atmosphere, the feedback exercises, the degree of student involvement, the comprehensibility and clarity of the content, the quality of the teaching, and overall satisfaction on a five-point Likert scale from 1 - very satisfied, 2 - quite satisfied, 3 - satisfied, 4 - rather dissatisfied, to 5 - dissatisfied. Additionally, two items on the questionnaire assess the respondents’ level of interest in interprofessionalism (“How interested are you in interprofessionalism?”) and their anticipated importance of team competencies in future medical practice (“How important are team competencies likely to be in your future medical practice?”). These are measured on a four-point Likert scale, ranging from 1 - not interested/ not important at all, 2 - some, 3 - moderate, to 4 - very interested/ very important.

### Data analysis

All analyses were performed with the jamovi software (version 2.3.28.0). Descriptive statistics (median, frequencies, mean and standard deviation) were calculated to describe the socio-demographics of the sample as well as levels of satisfaction with different aspects of the teaching unit.

To address the research questions, a linear regression analysis was conducted to examine how demographic variables (age and gender), interest in interprofessionalism, and perceived importance of interprofessionalism influence overall satisfaction with the seminar and perceived learning outcomes in theory and practice. The dependent variables included overall satisfaction with the seminar, which was measured on a 5-point Likert scale, as well as perceived learning outcome, which was also measured on a 5-point scale. This scale aligns with the familiar grading system and captures more nuanced or neutral responses. The independent variables consisted of age (measured as a metric), gender (dichotomous, with male as the reference category and female as the alternative), interest in interprofessionalism, and the perceived importance of interprofessionalism (4-point Likert scale). Because the items assess normative attitudes regarding interest and importance, a four-point Likert scale without a neutral midpoint was chosen. This encourages respondents to take a clear position. Thus, the scale was better suited to capturing decisive opinions on the topic.

The multiple linear regression models were constructed in a stepwise manner. In the initial model, age and gender were incorporated as independent variables to assess their impact on the dependent variables. In the second model, interest in interprofessionalism and the perceived importance of interprofessionalism were also included to ascertain their additional explanatory power with respect to the dependent variables. The quality of the model was evaluated using the adjusted coefficient of determination and F-tests of the overall model. Model comparisons were conducted using the difference in the explained variance (ΔR²) and significance tests. The regression coefficients (β), standardized effects and p-values were interpreted for the individual predictors. The standardized coefficients (ß) allow for the comparison of effect sizes across variables, independent of their original scale. Effects are considered statistically significant at *p* < 0.05.

## Results

### Sample characteristics

One hundred ninety-seven students took part in the learning unit. Of a total of 197 questionnaires issued, 192 were completed (corresponding to a 97.46% response rate). The age of the participants ranged from 20 to 34 years (M = 23.8 years, SD = 3.5 years). There were 57 male (corresponds to 29.8%, M = 23.8 years, SD = 2.91 years) and 134 female (corresponds to 70.2%, M = 24.0 years, SD = 3.15 years) participants, one person did not specify their gender.

### Descriptive results

**Fig. 1:**
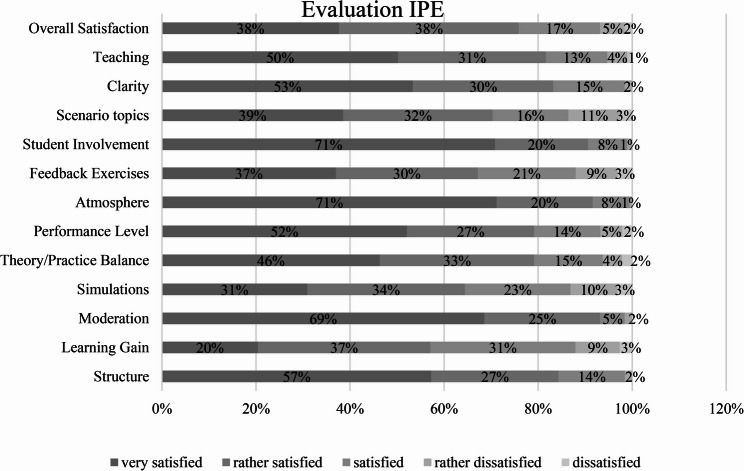
Evaluation of the IP learning unit

Overall, the participants were quite satisfied with the seminar (*M* = 1.95, *SD* = 0.95). Only 7% (*N* = 13) stated that they were rather dissatisfied or dissatisfied with the teaching unit. In particular, the moderation (69%), the atmosphere (71%) and the involvement of the students (71%) were rated as very satisfactory (Fig. 1). However, the participants were also very satisfied in the areas of structure, clarity, performance level and the teaching of the subject matter. The greatest variance was seen in the evaluation of the simulation-based learning scenarios, the selection of simulation-based learning scenarios topics and the feedback exercises. Overall, these were rated as highly satisfactory, as was the learning outcome from the teaching unit (Table 1).

**Table 1:** Descriptive statistics of satisfaction with the learning module

	*N*	Missing	Mean	Median	SD
Structure	192	0	1.60	1	0.786
Moderation	191	1	1.40	1	0.664
Simulation-based learning scenarios	191	1	2.21	2	1.08
Theory/Practice Balance	192	0	1.83	2	0.969
Performance Level	192	0	1.78	1	0.996
Atmosphere	191	1	1.38	1	0.652
Feedback Exercises	192	0	2.11	2	1.10
Student Involvement	192	0	1.40	1	0.686
Sceanrio Topics	192	0	2.07	2	1.10
Clarity	191	1	1.65	1	0.793
Teaching	191	1	1.74	1	0.913
Learning Outcomes	191	1	2.37	2	0.996
Overall Satisfaction	191	1	1.95	2	0.950

**Fig. 2:**
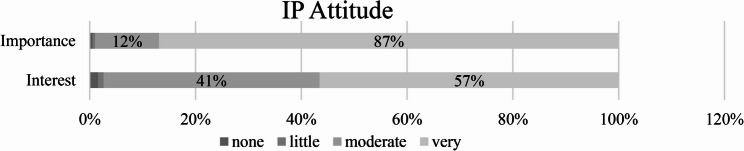
Interprofessional attitudes (Importance and Interest) of the students participants

87% of medical students rated team competence as very important for future medical practice (*N* = 166, M = 3,85, SD = 0,410). Interest in interprofessionalism was high among 57% (*N* = 108, M = 3,52, SD = 0,605) of students, while only 3% (*N* = 5) of the participants indicated little or no interest in the topic (Fig. 2).

### Impact on overall satisfaction with the learning module

Multiple linear regression analyses were conducted to examine the relationships between students’ individual characteristics (age, gender) and motivational factors (interest in interprofessionalism and the anticipated importance of interprofessional collaboration in future medical practice) with the overall satisfaction of the learning module (Table 2). The regression model that included both demographic and motivational variables significantly improved the explanation of variance in overall satisfaction, accounting for 12.66% of the total variance (*R²*= 0.1266, *F*(4,177) = 6.41, *p* < 0.001).

**Table 2:** Model fit statistics for the regression analysis

Overall Model Test								
Model	Predictor	*R*	*R*²	Adjusted *R*²	F	df1	df2	*p*
Overall Satisfaction	Age, Gender	0.133	0.0176	0.00664	1.60	2	179	0.204
Model Overall Satisfaction	Interest, Importance, Age, Gender	0.356	0.1266	0.10684	6.41	4	177	< 0.001
Model Learning Outcomes	Interest, Importance	0.336	0.113	0.108	23.9	1	188	< 0.001

As shown in Table 3, interest in interprofessionalism emerged as the only significant predictor of overall satisfaction (β= − 0.337, *p* < 0.001). The negative regression coefficient indicates that higher levels of interest were associated with greater satisfaction with the learning module, as reflected by lower values on the satisfaction scale.

**Table 3:** Model coefficients for the regression analysis

Predictor	Estimate b	SE	t-value	*p*	ß
Model: Overall satisfaction with the learning module					
Age	-0.0224	0.0220	-1.019	0.310	-0.0725
Gender	-0.1023	0.1448	-0.706	0.481	-0.1090
Interest	-0.5175	0.1110	-4.662	< 0.001	-0.3367
Importance	0.0476	0.1649	0.289	0.773	0.0212
Model: Satisfaction with learning outcomes in theory and practice					
Interest	-0,533	0.133	-4.88	< 0.001	-0.336
Importance	0.126	0.171	0.738	0.462	0.0519

*b*  unstandardized coefficient, *SE*  standard error, ß standardized coefficient, Gender coded as f = 1, m = 0

### Impact on satisfaction with learning outcomes in theory and practice

A separate multiple regression analysis was conducted to assess the impact of interest in interprofessionalism and anticipated importance of interprofessional collaboration on students’ satisfaction with the learning outcomes in theory and practice (Table 2). This model demonstrated a significant explanatory effect, accounting for 11.3% of the variance in satisfaction (R²= 0.113, F(1,188) = 23.9, *p* < 0.001).

Consistent with the first analysis, only interest in interprofessionalism significantly predicted satisfaction with learning outcomes (β= − 0.336, *p* < 0.001), indicating that students with higher levels of interest reported greater satisfaction with the learning outcomes in theory and practice, again reflected by lower values on the scale (Table 3).

## Discussion

The objective of this study was to evaluate a recently implemented interprofessional learning module that focuses on collaborative competencies through simulation-based scenarios and feedback. The findings contribute to the continuous integration of IPE in medical curricula in Germany, aiming to improve patient care in the face of evolving medical challenges.

The results indicate that the interprofessional learning unit was generally well received, and satisfaction with the teacher’s moderation, learning atmosphere, and active student engagement was rated highly. These aspects, and additionally student participation, are crucial for the success of interprofessional education formats, as supported by previous studies [17–19]. The focus on active student participation may have further contributed to higher levels of engagement and satisfaction, underscoring the importance of active learning strategies in interprofessional education [20].

Despite these positive findings, certain areas of the module revealed weaknesses that warrant further improvement. For instance, simulation-based learning scenarios, the selection of scenario topics, and feedback exercises exhibited great variability in student ratings. These results suggest potential areas for improvement. Previous studies highlight that the quality of simulation scenarios and the relevance of topics to clinical practice are critical factors for the effectiveness of IPE [21]. Additionally, structured, timely, and constructive feedback is known to be essential for maximizing learning outcomes [22, 23]. To enhance the module’s impact, future iterations should align simulation topics with students’ clinical interests and improve the feedback process. Adjustments have already been made to ensure the simulations are more representative of clinical practice, and video feedback will be introduced for more detailed, reflective learning [24, 25].

The study also revealed that a large proportion of students (87%) highly value teamwork in their future medical roles, and 57% have a strong interest in interprofessional collaboration. These results highlight the relevance of IPE in preparing students for collaborative practice and suggest that the module contributes significantly to fostering an interprofessional attitude. This aligns with the increasing recognition of teamwork as a core competency in healthcare education [6]. However, a minority of students (3%) expressed little or no interest in inteprofessionalism, indicating challenges in engaging all participants equally. Since interest in interprofessionalism significantly influenced overall satisfaction with the seminar and satisfaction with perceived learning outcomes in theory and practice, the question arises as to how this interest might be strategically enhanced. However, we cannot rule out the possibility that students with a preexisting interest in IPE were more likely to rate the module favorably. This limitation is discussed in more detail below.

### Perspectives for further development

A promising approach to promoting learning success lies in increasing situational interest. The hypothesis that repeated experiences in a learning environment that arouses situational interest could also lead to individual interest is supported by empirical evidence. To achieve this goal, different phases of interest development should be specifically addressed [26].

Firstly, there is considerable potential for the optimization of situational factors, with the aspects of novelty and surprise playing a decisive role in this process. One promising approach involves the integration of interprofessional teaching modules with other students of healthcare professions into the existing medical teaching curriculum. The introduction of such modules, which have been lacking to date, has the capacity to not only increase students’ situational interest but also to create more realistic simulation scenarios. These modules hold particular appeal for students with a strong clinical-practical focus and have the potential to establish a more robust link between interprofessional collaboration and individual interest.

In addition, the integration of interprofessional, problem-based learning (PBL) could sustainably promote students’ interest [27, 28]. The relevance of interprofessional collaboration is clearly emphasized through the joint analysis and discussion of patient cases from different professional perspectives. This experience underscores the significance of interprofessional approaches to medical care, fostering long-term interest and commitment among students. To support this process, it is essential to establish designated tasks and times for targeted reflection. One potential approach to this implementation could be the incorporation of a reflection report on (inter-)professional attitudes in an earlier segment of the course.

A further approach to be considered would be the establishment of interprofessional training wards. In such wards, students from different healthcare professions could collaborate under supervision in a practical setting and address authentic, complex problems. This approach would not only facilitate a more profound comprehension of the roles of other professions, but also cultivate teamwork skills under realistic conditions and foster interest in interprofessional collaboration at a higher level of difficulty. This method has been identified as promising in IPE [29–31].

Finally, even if the anticipated importance of interprofessionalism was already high, the value of the interprofessional approach could be strengthened by promoting a culture of interprofessional collaboration within the educational institution [26]. The reduction of hierarchies, the establishment of shared learning spaces, and the formation of interprofessional networks have the potential to enhance student interest and motivation in interprofessional collaboration and help to form an interprofessional attitude and thinking.

Furthermore, future research building on the findings of this study would benefit from systematically evaluating the effectiveness of the modules in fostering learning outcomes and attitudinal change. This could be achieved through pre- and post-assessments, as well as knowledge and skills evaluations. Commonly used instruments for assessing interprofessional learning and collaboration include the Readiness for Interprofessional Learning Scale (RIPLS) developed by Parsell and Bligh [32] and revised by McFadyen et al. [34, 35], which measures readiness for IPE, and the Interdisciplinary Education Perception Scale (IEPS) published by Luecht et al. (1990), which focuses on attitudes and skills [32–35]. Both are widely recognized and frequently employed in IPE research [36–38].

## Limitations of the study

This study has several limitations that restrict the interpretation of the results. Firstly, no previous professional experience in the healthcare sector or other educational backgrounds of the participants were recorded. This makes it difficult to consider possible prior knowledge and its influence on the results. Secondly, the inclusion of only medical students may limit the generalizability of the findings to an interprofessional education context. Despite the recognized efficacy of simulation and role-play as educational methodologies, these approaches may not fully capture the complexity of real-world team dynamics, especially because the concept lacks an authentic multi-professional interaction, which could have limited participants’ ability to value team-based competencies from diverse professional perspectives.

Additionally, as no pre-/post-design was employed we cannot infer causality. It is possible that students who were already interested in IPE evaluated the module more positively, rather than the module itself increasing interest. Furthermore, the presence of a ceiling effect in the questionnaire may have led to an underestimation of differences or improvements in responses. Moreover, no validated and widely established measurement instrument for IPE was employed in this study. Since the primary focus of the present evaluation was on the overall module - encompassing multiple perspectives, including didactic and organizational aspects - the assessment centered on the general teaching concept rather than explicitly measuring interprofessional learning outcomes. In subsequent research, greater emphasis should be placed on evaluating the development of interprofessional competencies. However, not all established IPE measurement instruments are currently available in German or have been validated according to psychometric standards [39, 40]. Validated German-language instruments such as the University of the West of England Interprofessional Questionnaire – German Version (UWE-IP-D) and the German Interprofessional Attitudes Scale (G-IPAS) represent promising tools for this purpose [41, 42]. Future studies should consider incorporating such standardized instruments, ideally in combination with performance-based or behavioral measures, to enhance the validity, reliability, and comparability of outcome assessments in interprofessional education research.

## Conclusion and implications

This study provides valuable insights into the implementation and evaluation of an interprofessional learning module aimed at enhancing collaborative competencies through role-playing and feedback. The results demonstrate the module’s effectiveness in fostering an interprofessional attitude, supported by high student satisfaction and strong interest in interprofessionalism. These findings highlight the potential of IPE to equip students with critical teamwork and collaboration skills for modern healthcare.

A key strength of the present study is the systematic combination of multiple pedagogically validated IPE methods and transparent documentation of their implementation within the formal medical curriculum. This contrasts with much of the existing IPE literature, in which the curricular context, methodological design, and timing of interventions are often inadequately described, thereby limiting reproducibility and comparability across studies [14].

Furthermore, integrating multiple theory-based learning formats, ranging from lectures to simulation-based seminars with structured debriefing, highlights the importance of a multimodal approach to developing interprofessional competencies. Future studies aiming to assess the sustained impact of IPE on learner outcomes and collaborative behavior in clinical settings may benefit from adopting similarly structured and well-documented interventions.

However, variability in ratings of simulation scenarios and feedback exercises suggests room for improvement. Aligning simulation topics with clinical interests and incorporating video feedback could enhance the module’s impact. Integrating problem-based learning and training wards may also strengthen students’ collaborative skills.

Despite the module’s success, some students displayed low interest in IPE, emphasizing the need for strategies to engage all participants. Enhancing situational interest with novel elements and structural changes like reducing hierarchies and creating shared learning spaces may boost the perceived value of IPE.

Ultimately, continuous refinement and integration of interprofessional learning modules into medical curricula hold significant promise for improving patient care by preparing future healthcare professionals for effective collaboration in a complex medical environment.

## Supplementary Information

Supplementary Material 1.

## Data Availability

The raw data from this study is available upon request from the corresponding author.
