# Supplementary material for: Evaluation of a learning module on team competencies in medicine utilizing simulation-based scenarios
Source: BMC Med Educ. 2026 Apr 24;26:675. doi: 10.1186/s12909-026-09274-9 (PMC13107668; doi:10.1186/s12909-026-09274-9)
Supplement: Supplementary file 1 — Supplementary Material 1. [file 12909_2026_9274_MOESM1_ESM.docx]

**Evaluation Learning module - team competencies**

| **Date** | __ __ __ __ 2024 |  |  | **ID**KPSM |  |
| --- | --- | --- | --- | --- | --- |
| **Gender** | ❑0 male ❑1 female ❑2 other | | | **age** | ____ years |

| ***satisfaction with the learning module*:** | very satisfied | rather satisfied | satisfied | rather dissatisfied | dissatisfied |
| --- | --- | --- | --- | --- | --- |
| Structure | ❑1 | ❑2 | ❑3 | ❑4 | ❑5 |
| Learning outcome | ❑1 | ❑2 | ❑3 | ❑4 | ❑5 |
| Moderation | ❑1 | ❑2 | ❑3 | ❑4 | ❑5 |
| Simulation-based learning scenarios | ❑1 | ❑2 | ❑3 | ❑4 | ❑5 |
| Theory/Practice Balance | ❑1 | ❑2 | ❑3 | ❑4 | ❑5 |
| Performance level | ❑1 | ❑2 | ❑3 | ❑4 | ❑5 |
| Atmosphere | ❑1 | ❑2 | ❑3 | ❑4 | ❑5 |
| Feedback exercises | ❑1 | ❑2 | ❑3 | ❑4 | ❑5 |
| Student involvement | ❑1 | ❑2 | ❑3 | ❑4 | ❑5 |
| Scenario topics | ❑1 | ❑2 | ❑3 | ❑4 | ❑5 |
| Clarity | ❑1 | ❑2 | ❑3 | ❑4 | ❑5 |
| Teaching | ❑1 | ❑2 | ❑3 | ❑4 | ❑5 |
| **Overall satisfaction** | ❑1 | ❑2 | ❑3 | ❑4 | ❑5 |

| **How interested are you in interprofessionalism?** | | | | | |
| --- | --- | --- | --- | --- | --- |
| ❑1 not interested | ❑2 some | ❑3 moderate | ❑4 very interested | |  |
|  |  |  |  |  | |

| **How important are team competencies likely to be in your future medical practice?** | | | | | | | |
| --- | --- | --- | --- | --- | --- | --- | --- |
| ❑1 not important at all | | ❑2 some | | ❑3 moderate | ❑4 very important | |  |
|  |  | |  | |  |  | |
